# Oromandibular dystonia unresponsive to botulinum toxin: two illustrative cases of condylar dislocation as a structural mimic

**DOI:** 10.3389/fneur.2026.1912300

**Published:** 2026-07-20

**Authors:** Olcay Şakar, Berk Bilgen, Bora Edim Akalın, Kazuya Yoshida

**Affiliations:** 1 Department of Prosthodontics, İstanbul University Faculty of Dentistry, İstanbul, Türkiye; 2 Department of Plastic, Reconstructive and Aesthetic Surgery, Istanbul University Faculty of Medicine, İstanbul, Türkiye; 3 Department of Oral and Maxillofacial Surgery, National Hospital Organization Kyoto Medical Center, Kyoto, Japan

**Keywords:** botulinum toxin therapy, differential diagnosis, oromandibular dystonia, structural mimic, temporomandibular joint dislocation, treatment non-response

## Abstract

Botulinum toxin therapy is a mainstay treatment for oromandibular dystonia (OMD), but lack of response should prompt reconsideration of both the injection strategy and the underlying diagnosis. Long-standing condylar dislocation is a structural disorder that can mimic OMD by producing mandibular deviation, impaired mandibular closure, dysarthria, dysphagia, and abnormal jaw or tongue movements. We present two illustrative cases in which patients initially diagnosed with OMD underwent botulinum toxin injections into the masseter muscles without meaningful benefit. Subsequent dental and oral/maxillofacial evaluation, including panoramic radiography, revealed long-standing condylar dislocation as the primary diagnosis. Both patients improved after structural correction and prosthetic rehabilitation. These cases do not establish masseter injection failure as diagnostic evidence against OMD; rather, they highlight botulinum toxin non-response as a practical clinical trigger for systematic reassessment in selected patients with suspected OMD. In patients with fixed mandibular deviation, persistent occlusal disturbance, impaired closure, facial asymmetry, or atypical dystonic features, structural reassessment with dental imaging and oral/maxillofacial consultation should be considered before continuing chemodenervation.

## Introduction

Dystonia is defined by sustained or intermittent muscle contractions causing abnormal, often repetitive movements, postures, or both (1). Oromandibular dystonia (OMD) is a focal dystonia involving the masticatory, lower facial, labial, and/or lingual muscles. Depending on the direction and distribution of abnormal movements, OMD may present as jaw-closing, jaw-opening, jaw-deviation, jaw-protrusion, lingual, or lip dystonia. Large clinical series and systematic reviews have emphasized the heterogeneity of OMD, including impaired mastication, dysarthria, dysphagia, mandibular deviation, tongue involvement, orofacial pain, and temporomandibular joint (TMJ)-related symptoms (2–7).

Botulinum toxin injection is an established treatment for OMD when the responsible muscles are appropriately identified (5, 6, 8). However, non-response to botulinum toxin therapy should not be interpreted solely as pharmacological treatment resistance. It may also reflect inappropriate muscle selection, insufficient phenomenological characterization, coexisting disorders, or an alternative non-dystonic diagnosis. This is particularly important in OMD-like presentations involving jaw deviation or impaired mandibular closure, where structural disorders of the TMJ and occlusion may resemble dystonic movements (8–11).

The clinical diagnosis of OMD is supported by features typical of focal dystonia, including stereotypy, task specificity, sensory tricks, overflow phenomenon, morning benefit, and co-contraction (1, 2, 8, 10). The presence of multiple such features supports the diagnosis, whereas their absence should prompt careful reconsideration. In contrast, TMJ dislocation is a structural condition in which the mandibular condylar head is displaced beyond the articular eminence and cannot be self-reduced. Etiologic factors include muscle hyperactivity, ligamentous laxity, steep articular eminence, abnormal condylar morphology, neurological disease, and systemic hypermobility (12–15). When unreduced for a prolonged period, condylar dislocation becomes long-standing and is less likely to respond to manual reduction or conservative treatment (13–18).

The distinction between OMD and long-standing condylar dislocation has received limited attention despite their overlapping clinical features. Both may present with mandibular deviation, impaired closure, facial asymmetry, speech disturbance, swallowing difficulty, and movements that appear stereotyped or co-contractive (2, 9, 10, 18). However, the therapeutic implications differ fundamentally. OMD requires neurological assessment and appropriately targeted chemodenervation, whereas long-standing condylar dislocation requires structural evaluation and, in selected cases, oral/maxillofacial or prosthetic management (8, 12–18).

Here, we present two illustrative cases in which patients with OMD-like symptoms underwent botulinum toxin injections without meaningful benefit before dental and oral/maxillofacial evaluation revealed long-standing condylar dislocation. The cases are used as a paradigm to discuss botulinum toxin non-response as a prompt for diagnostic reassessment and to propose practical diagnostic clues for neurologists and movement disorder clinicians.

These two cases were selected as illustrative examples because they shared a similar diagnostic trajectory: an initial diagnosis of OMD, ineffective botulinum toxin treatment, subsequent dental/oral-maxillofacial assessment, and final diagnosis of long-standing condylar dislocation. They are not intended to represent the frequency or full clinical spectrum of structural mimics among patients with suspected OMD, and selection bias is inherent in this case-based Perspective.

## Illustrative cases

### Case 1

A 65-year-old edentulous woman presented with inability to achieve complete mandibular closure, mandibular deviation, intermittent involuntary jaw contractions, and orofacial pain. Approximately 15 years earlier, she had developed pain in the right preauricular and masseteric region during a period of severe emotional stress, followed by progressive limitation of jaw movement and facial asymmetry. During the preceding 3 years, she had experienced near-complete inability to close her jaw, rightward mandibular deviation, severely restricted mandibular movements, and intermittent jaw contractions during speech.

She had been evaluated in several medical departments and was eventually diagnosed with OMD after neurological assessment. Multiple sessions of botulinum toxin injections into the masseter muscles were performed, but no clinically meaningful improvement was obtained. These injections had been performed before referral, and detailed treatment records regarding dose, formulation, exact injection sites, and use of EMG or ultrasound guidance were not available.

Dental examination revealed marked facial asymmetry, mandibular deviation to the right, bilateral TMJ tenderness, severely restricted eccentric movements, impaired mandibular closure, and abnormal tongue control during speech (Figure 1A). Panoramic radiography demonstrated bilateral anterior displacement of the condylar heads beyond the articular eminences, consistent with long-standing bilateral condylar dislocation (Figure 1B). Manual reduction was unsuccessful.

**Figure 1:**
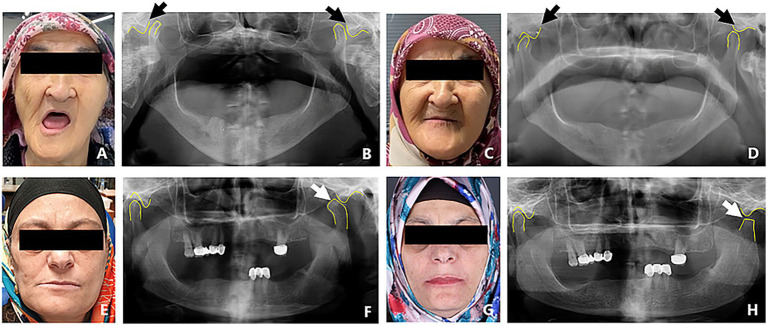
Clinical and radiographic findings in two illustrative cases of long-standing condylar dislocation mimicking oromandibular dystonia. **(A–D)** Case 1. **(A)** Preoperative frontal view showing mandibular deviation and facial asymmetry secondary to bilateral long-standing condylar dislocation. **(B)** Panoramic radiograph showing bilateral anterior displacement of the condylar heads beyond the articular eminences; arrows indicate the bilateral articular eminences. **(C)** Postoperative frontal view showing partial resolution of facial asymmetry and improved mandibular position. **(D)** Postoperative panoramic radiograph showing the position of the bilateral condylar heads after open eminectomy and capsular stabilization. **(E–H)** Case 2. **(E)** Preoperative frontal view showing mandibular deviation to the right and facial asymmetry. **(F)** Preoperative panoramic radiograph showing anterior displacement of the left mandibular condylar head with associated hyperplastic changes; the arrow indicates the hyperplastic condylar head. **(G)** Postoperative frontal view after surgical and prosthetic rehabilitation showing resolution of mandibular deviation and restoration of mandibular symmetry. **(H)** Postoperative panoramic radiograph after high condylectomy showing the surgically remodeled condylar region; the arrow indicates the corrected condylar area.

Because the dislocation was chronic and structural obstruction by prominent articular eminences was suspected, she was referred for oral/maxillofacial surgical management. Bilateral open eminectomy with capsular stabilization was performed (19). Postoperatively, mandibular closure, facial asymmetry, and jaw movements improved markedly (Figures 1C,D). Mild residual involuntary jaw movements persisted, raising the possibility of a concomitant dystonic component or secondary motor adaptation.

### Case 2

A 64-year-old woman with a history of stroke 1 year earlier presented with mandibular asymmetry, difficulty achieving occlusal contact, impaired mastication, and speech disturbance. She had previously been evaluated by neurologists and given a provisional diagnosis of OMD. A single session of botulinum toxin injection into the masseter muscles was performed without meaningful improvement. Detailed records regarding dose, formulation, injection sites, and use of EMG or ultrasound guidance were not available.

Clinical evaluation showed rightward mandibular deviation, facial asymmetry, marked restriction of mandibular movement, inability to achieve stable bilateral occlusal contact, difficulty with speech and swallowing, and abnormal tongue movements during speech (Figure 1E). The tongue movements were considered secondary to the altered mandibular position rather than primary lingual dystonia. Panoramic radiography demonstrated anterior and inferior displacement of the left mandibular condyle with hyperplastic changes of the condylar head, indicating long-standing unilateral condylar dislocation associated with condylar hyperplasia (Figure 1F).

Manual reduction was unsuccessful, and the patient underwent high condylectomy with disc repositioning, followed by prosthetic rehabilitation (17). The patient regained stable mandibular closure and bilateral occlusal contact, with resolution of facial asymmetry and abnormal tongue movements during speech (Figures 1G,H). At long-term follow-up, symptoms remained resolved without recurrence.

### Why condylar dislocation can mimic OMD

Long-standing condylar dislocation and OMD are etiologically distinct but may overlap clinically. OMD is a neurological disorder characterized by involuntary patterned muscle contractions, whereas condylar dislocation is a mechanical or anatomical disorder of the TMJ (1–7, 12–15). Nevertheless, both conditions may produce mandibular deviation, impaired closure, facial asymmetry, speech disturbance, swallowing difficulty, and abnormal orofacial movements. This overlap can make symptom-based differentiation unreliable (2, 9, 10, 18).

Among OMD subtypes, jaw-opening and jaw-deviation dystonia most closely resemble condylar dislocation (2, 6, 8). A fixed mandibular position in long-standing dislocation may appear stereotyped because the deviation and impaired closure are consistent across repeated attempts. However, this apparent stereotypy reflects the fixed displacement of the condylar head rather than patterned dystonic activation. Similarly, reflexive masticatory muscle spasm secondary to condylar displacement may mimic co-contraction, but the mechanism is mechanical restriction rather than involuntary simultaneous activation of antagonist muscles (12–15, 18).

Several features may help distinguish these conditions. Primary OMD is often characterized by task specificity, sensory tricks, overflow phenomenon, morning benefit, and variable dystonic activation (1, 2, 8, 10). By contrast, long-standing condylar dislocation tends to produce persistent symptoms that remain relatively constant regardless of task, time of day, or sensory input. Fixed occlusal disturbance, inability to achieve stable mandibular closure, persistent anterior open bite or unilateral occlusal discrepancy, and radiographic displacement of the condylar head are particularly important clues (12–18) (Table 1).

**Table 1:** Differential diagnostic features of oromandibular dystonia and long-standing condylar dislocation.

Finding	Oromandibular dystonia	Long-standing condylar dislocation
Etiology	Neurological disorder characterized by involuntary dystonic muscle contractions	Mechanical or anatomical disorder of the temporomandibular joint
Radiographic findings	Usually normal TMJ anatomy, unless secondary changes coexist	Displacement of the condylar head beyond the articular eminence on panoramic radiography or cone-beam computed tomography
Mandibular findings	Vary according to subtype; may include jaw closing, jaw opening, jaw deviation, jaw protrusion, lingual, or lip involvement	Persistent inability to achieve complete mandibular closure, mandibular deviation, unstable occlusion, and/or anterior open bite depending on the type and duration of dislocation
Stereotypy	Typically present	May appear present because mandibular deviation and restricted closure are consistent, but this reflects fixed condylar displacement rather than patterned dystonic activation
Task specificity	Often present	Usually absent; symptoms are persistent and not limited to specific tasks
Sensory trick	May be present	Absent; sensory stimulation does not resolve the mechanical displacement
Symptoms upon awakening	Morning benefit may be present	Usually absent; symptoms remain relatively constant regardless of time of day
Co-contraction	May be present	May mimic co-contraction because condylar displacement can trigger reflexive masticatory muscle spasm and mechanical restriction
Overflow phenomenon	May be present	Usually absent
Botulinum toxin response	May respond when the correct dystonic muscles are identified and appropriately injected	Usually ineffective for long-standing structural dislocation, although botulinum toxin may be useful in recurrent or neurogenic dislocation driven by muscle hyperactivity
Diagnostic implication	Supported by typical dystonic features, electromyographic findings, and response to appropriately targeted treatment	Suspected when fixed mandibular deviation, impaired closure, persistent occlusal disturbance, facial asymmetry, or radiographic condylar displacement is present

OMD, oromandibular dystonia; TMJ, temporomandibular joint.

### Botulinum toxin non-response as a diagnostic red flag

In both cases, botulinum toxin injections were administered to the masseter muscles without meaningful clinical benefit. This observation requires a cautious interpretation.

Importantly, the absence of clinical improvement after masseter injection should not be interpreted as diagnostic evidence against OMD. In jaw-opening or jaw-deviation dystonia, the masseter is not usually the principal therapeutic target, and careful evaluation of the lateral pterygoid and other involved muscles may be required (5, 6, 8, 20). Thus, in the present cases, non-response to masseter injection may have reflected inappropriate muscle selection, the presence of a structural mimic, or both. The clinical value of this observation is therefore not that masseter injection failure proves condylar dislocation, but that treatment non-response in an atypical OMD-like presentation should trigger systematic reassessment of the phenomenology, injection strategy, and structural differential diagnosis.

Botulinum toxin injection can be useful in recurrent or neurogenic TMJ dislocation when excessive muscle activity contributes to repeated displacement (14, 20). However, in long-standing dislocation caused by structural factors such as prominent articular eminence or condylar hyperplasia, chemodenervation alone is unlikely to correct the underlying mechanical abnormality (12–18). In such cases, continuing botulinum toxin therapy without structural reassessment may delay appropriate diagnosis and treatment.

Therefore, botulinum toxin non-response in suspected OMD should be regarded as a clinical red flag only in the broader clinical context, particularly when accompanied by fixed mandibular deviation, persistent inability to close the jaw, occlusal instability, facial asymmetry, or atypical dystonic features. The clinician should reconsider not only dose and target muscle, but also the possibility of a structural mimic (1, 2, 8–11, 18).

### Practical implications for neurologists and movement disorder clinicians

The key issue for neurologists is not how to surgically manage condylar dislocation, but when to suspect it. In practical terms, dental imaging should be considered when suspected OMD is accompanied by one or more structural warning signs: fixed mandibular deviation, persistent inability to achieve complete mandibular closure, anterior open bite or unstable occlusion, unilateral occlusal discrepancy, TMJ pain or tenderness, facial asymmetry, or radiographic suspicion of condylar malposition. The threshold for referral should be particularly low when these findings coexist with poor response to appropriately planned botulinum toxin therapy or when typical dystonic features, such as task specificity, sensory tricks, overflow phenomenon, or morning benefit, are absent (8–15, 18, 20).

Panoramic radiography and, when necessary, cone-beam computed tomography can identify condylar displacement, abnormal articular eminence morphology, condylar hyperplasia, or other structural abnormalities (12–18). In both illustrative cases, the correct diagnosis was established by routine radiographic assessment after neurological diagnosis and ineffective botulinum toxin therapy. This highlights the importance of integrating dental imaging into the diagnostic pathway for selected patients with OMD-like presentations.

Importantly, OMD and structural TMJ pathology should not be regarded as mutually exclusive. Long-standing dystonic activity may contribute to secondary TMJ symptoms, while chronic mechanical stress from dislocation may induce compensatory or secondary motor patterns that resemble dystonia (9, 18, 20). In mixed cases, clinicians should avoid a binary diagnostic approach. Instead, neurological phenomenology, EMG when available, dental occlusal assessment, and TMJ imaging should be integrated to determine whether chemodenervation, structural treatment, prosthetic rehabilitation, or combined management is most appropriate. The presence of residual abnormal movements after structural correction, as observed in Case 1, suggests that mixed mechanisms may occasionally be present. A collaborative approach involving neurology, dentistry, and oral/maxillofacial surgery is therefore essential.

### Future directions

Future studies should clarify how frequently structural TMJ disorders mimic OMD in clinical practice and which clinical features best predict the need for dental imaging. Although large clinical series have described the heterogeneous phenomenology of OMD, data specifically quantifying misdiagnosis between OMD and structural TMJ disorders remain limited. Prospective evaluation of patients with suspected OMD who fail botulinum toxin therapy may help define a practical diagnostic algorithm. Incorporating standardized OMD clinical criteria, EMG, dental examination, and TMJ imaging could improve diagnostic accuracy and reduce unnecessary or ineffective treatment (2, 6, 8, 10, 18).

## Limitations

These illustrative cases have important limitations. They were not evaluated within a prospective research protocol, and standardized OMD rating scales, EMG, or blinded video review were not performed. Therefore, the presence of subtle concomitant dystonia cannot be completely excluded, particularly in Case 1, in whom mild residual involuntary jaw movements persisted after structural correction. Detailed botulinum toxin injection records were unavailable; therefore, we cannot determine whether the lack of response reflected suboptimal target selection, inadequate dosing or guidance, the structural nature of the underlying disorder, or a combination of these factors. Quantitative outcome measures, including maximal jaw opening, occlusal contact counts, validated chewing or speech questionnaires, and standardized pre/post imaging measurements, were not available. Post-treatment outcomes were therefore described based on clinical follow-up observations and patient-reported improvement. Accordingly, we interpret botulinum toxin non-response not as diagnostic proof of condylar dislocation, but as a practical clinical red flag that should prompt reassessment of the phenomenology, injection plan, and structural differential diagnosis.

### Conclusion

Long-standing condylar dislocation can present as a structural mimic of OMD and may lead to ineffective botulinum toxin therapy if dental and oral/maxillofacial evaluation is not incorporated into the diagnostic process. In patients with suspected OMD, lack of response to botulinum toxin should prompt reassessment of both the injection strategy and the underlying diagnosis, rather than being interpreted as diagnostic evidence by itself. Fixed mandibular deviation, impaired closure, persistent occlusal disturbance, facial asymmetry, and absence of typical dystonic features should raise suspicion for a structural mimic. Early interdisciplinary evaluation and appropriate imaging may prevent diagnostic delay and guide effective treatment.

## Data Availability

The datasets presented in this article are not readily available because of ethical and privacy restrictions. Requests to access the datasets should be directed to the corresponding author.

## References

[1] Albanese A Bhatia K Bressman SB Delong MR Fahn S Fung VSC . Phenomenology and classification of dystonia: a consensus update. Mov Disord. (2013) 28:863–73. doi: 10.1002/mds.25475, 23649720 PMC3729880

[2] Scorr LM Factor SA Parra SP Kaye R Paniello RC Norris SA . Oromandibular dystonia: a clinical examination of 2,020 cases. Front Neurol. (2021) 12:700714. doi: 10.3389/fneur.2021.700714, 34603182 PMC8481678

[3] Slaim L Cohen M Klap P Vidailhet M Perrin A Brasnu D . Oromandibular dystonia: demographics and clinical data from 240 patients. J Mov Disord. (2018) 11:78–81. doi: 10.14802/jmd.17065, 29860784 PMC5990905

[4] Raoofi S Khorshidi H Najafi M. Etiology, diagnosis and management of oromandibular dystonia: an update for stomatologists. J Dent (Shiraz). (2017) 18:73–81. 28620630 PMC5463774

[5] Comella CL. Systematic review of botulinum toxin treatment for oromandibular dystonia. Toxicon. (2018) 147:96–9. doi: 10.1016/j.toxicon.2018.02.006, 29453996

[6] Bhidayasiri R Maytharakcheep S Truong DD. Patient selection and injection techniques for botulinum neurotoxin in oromandibular dystonia. Clin Parkinsonism Relat Disord. (2022) 7:100160. doi: 10.1016/j.prdoa.2022.100160 PMC9399243 36033904

[7] Saraf U Sardana V Pant R Agarwal V Bhatia R Kumaran S . Oromandibular dystonia: a systematic review. Ann Indian Acad Neurol. (2022) 25:26–34. doi: 10.4103/aian.aian_242_21 35342238 PMC8954320

[8] Yoshida K. Botulinum toxin therapy for oromandibular dystonia and other movement disorders in the stomatognathic system. Toxins (Basel). (2022) 14:282. doi: 10.3390/toxins14040282, 35448891 PMC9026473

[9] Britton D Alty JE Mannion CJ. Oromandibular dystonia: a diagnosis not to miss. Br J Oral Maxillofac Surg. (2020) 58:520–4. doi: 10.1016/j.bjoms.2020.01.015 32143935

[10] Yoshida K. Oromandibular dystonia screening questionnaire for differential diagnosis. Clin Oral Investig. (2019) 23:405–11. doi: 10.1007/s00784-018-2449-3, 29717363

[11] Yoshida K. Bruxism or dystonia: that is the question. J Oral Maxillofac Surg. (2023) 81:935–7. doi: 10.1016/j.joms.2023.04.002 37536913

[12] Neff A McLeod N Spijkervet FKL Riechmann M Vieth U Kolk A . The ESTMJS (European Society of Temporomandibular Joint Surgeons) consensus and evidence-based recommendations on management of condylar dislocation. J Clin Med. (2021) 10:5068. doi: 10.3390/jcm10215068, 34768586 PMC8584777

[13] Abrahamsson H Eriksson L Abrahamsson P Haggman-Henrikson B. Treatment of temporomandibular joint luxation: a systematic literature review. Clin Oral Investig. (2020) 24:61–70. doi: 10.1007/s00784-019-03126-1, 31729577

[14] Prechel U Ottl P Ahlers OM Neff A. The treatment of temporomandibular joint dislocation. Dtsch Arztebl Int. (2018) 115:59–64. doi: 10.3238/arztebl.2018.0059, 29439762 PMC5817180

[15] Marques-Mateo M Puche-Torres M Iglesias-Gimilio ME. Temporomandibular chronic dislocation: the long-standing condition. Med Oral Patol Oral Cir Bucal. (2016) 21:e776–83. doi: 10.4317/medoral.21221, 27694782 PMC5116121

[16] Tanaka T Momozaki N Honda E Matsuno A. Delayed diagnosis of temporomandibular joint dislocation in severe stroke patients. Cureus. (2024) 16:e68896. doi: 10.7759/cureus.68896, 39376821 PMC11458201

[17] Uetsuki R Ono S Tada M Okuda S Takechi M. Long-standing temporomandibular joint dislocation treated by intraoral condylectomy: a case report and review of the literature. J Med Case Rep. (2022) 16:245. doi: 10.1186/s13256-022-03471-y 35733220 PMC9219198

[18] Yoshida K. Long-standing temporomandibular joint dislocation: a comprehensive review and proposal of a treatment algorithm. Medicina (Kaunas). (2025) 61:1505. doi: 10.3390/medicina61091505, 41010896 PMC12471310

[19] Sato J Segami N Nishimura M Suzuki T Kaneyama K Fujimura K. Clinical evaluation of arthroscopic eminoplasty for habitual dislocation of the temporomandibular joint: comparative study with conventional open eminectomy. Oral Surg Oral Med Oral Pathol Oral Radiol Endod. (2003) 95:390–5. doi: 10.1067/moe.2003.128, 12686922

[20] Yoshida K. Botulinum neurotoxin injection for the treatment of recurrent temporomandibular joint dislocation with and without neurogenic muscular hyperactivity. Toxins (Basel). (2018) 10:174. doi: 10.3390/toxins10050174, 29693593 PMC5983230

